# Draft Whole-Genome Sequence of *Triparma laevis* f. *inornata* (Parmales, Bolidophyceae), Isolated from the Oyashio Region, Western North Pacific Ocean

**DOI:** 10.1128/MRA.00367-20

**Published:** 2020-08-13

**Authors:** Akira Kuwata, Kenji Saitoh, Yoji Nakamura, Mutsuo Ichinomiya, Naoki Sato

**Affiliations:** a Tohoku National Fisheries Research Institute, Fisheries Research and Education Agency, Shiogama, Miyagi, Japan; b National Research Institute of Fisheries Science, Fisheries Research and Education Agency, Yokohama, Kanagawa, Japan; c Prefectural University of Kumamoto, Kumamoto, Kumamoto, Japan; d University of Tokyo, Tokyo, Japan; University of California, Riverside

## Abstract

We present the first draft whole-genome sequence for the Parmales (Bolidophyceae, Heterokonta), a picoplanktonic sister group of diatoms, using a *Triparma laevis* f. *inornata* strain that was isolated from the Oyashio region in the western North Pacific Ocean.

## ANNOUNCEMENT

The Parmales (Bolidophyceae) are a group of pico-sized marine phytoplankton with a silicified cell wall that are distributed widely in the world’s oceans, from polar to subtropical regions (1). Analysis of small-subunit ribosomal DNA and *rbcL* genes and the organellar genomes of *Triparma laevis* revealed that the Parmales are members of the Heterokonta and share a common ancestor with diatoms, which are the most successful phytoplankton in modern oceans (2, 3). The origin of the silica cell wall and the early evolution of diatoms are still unclear, and genomic analysis of Parmales may help address these issues. We present the first draft whole-genome sequence for the Parmales, that of a *Triparma laevis* f. *inornata* strain (NIES-2565) that was isolated from the Oyashio region of the western North Pacific Ocean.

The algal strain was cultured in f/2 medium (4) at 5°C under ∼30 μmol photons m^−2^ s^−1^ (14-h light/10-h dark photoperiod) (2). Cells in the exponential growth phase, ∼3 days after inoculation, were collected on polycarbonate filters (0.6-μm pore size; Millipore). DNA was extracted by treatment with sodium *N*-dodecanoylsarcosinate and proteinase K, extracted with phenol-chloroform, and purified by CsCl ultracentrifugation (3).

Preparation of sequence templates for 454 FLX+ (shotgun and paired-end) sequencing and Illumina GAII (paired-end) sequencing followed the manufacturers’ instructions using the Roche 454 GS FLX Titanium rapid library preparation kit and the Illumina TruSeq DNA PCR-free sample preparation kit, respectively. To obtain short (∼800-bp) fragments for 454 shotgun and Illumina paired-end sequencing, an S220 ultrasonicator (Covaris, Woburn, MA, USA) was used to shear genomic DNA. The HydroShear device (Digilab, Marlborough, MA, USA) was used to obtain longer (∼3-kb) fragments for 454 paired-end sequencing. We first assembled 454 genomic shotgun reads (∼2.75 million reads) and paired-end reads (∼2.27 million pairs of reads) (31.1× coverage overall) with the Newbler assembler (version 2.9; Roche Diagnostics). Newbler excluded about 15% of reads by filtering low-quality or highly repetitive reads. The paired-end insert length given by Newbler (∼1.6 kb) may be underestimated because of skew to shorter inserts with both reads mapped. For Illumina reads, low-quality end nucleotides were trimmed using a Perl script (34.4× coverage subsequently) before further analysis. Because 454 pyrosequencing reads inherently have frequent miscalls of the number of mononucleotide tracts (5), length disagreements were overridden by Illumina reads upon >8× coverage (1,602 sites) using an available Perl script (http://cse.fra.affrc.go.jp/ksaitoh/script/OverrideSeqs.pl). These length disagreements were detected by mapping Illumina paired-end reads (2 × 71 to 73 bp, with insert lengths of ∼800 bp) onto the 454 scaffolds by Bowtie 2 (version 2.1.0) (6). The improved 454 scaffolds and contigs were then bridged by Illumina paired-end reads with SSPACE-basic (version 2.0) (7) with the options -k 5 -a 0.7 -x 1 -m 50 -o 10 -t 12 -r 0.8. Gaps were filled with the Illumina reads by GapFiller (version 1.10) (8) with the options -m 30 -o 2 -r 0.7 -n 10 -d 50 -t 10 -g 0 -T 4 -i 1.

As a result, 13,538 contigs (*N*_50_, 15,437 bp) with 62,124,971 bp in total were obtained. Bridging these contigs yielded 2,930 scaffolds (≥500 bp) totaling 62,842,229 bp in length (*N*_50_, 89,610 bp), but some of those were derived from the plastid and mitochondrion of *T. laevis* (3) or bacteria in the culture. To clean these, the scaffolds were further classified based on 4-bp nucleotide frequency using principal-component analysis and t-SNE (9, 10). Finally, a total of 902 scaffolds were collected as those of the *T. laevis* nuclear genome, totaling 42,573,571 bp with an *N*_50_ value of 83,218 bp and a G+C content of 51.3%. The genome sequence quality was assessed by Benchmarking Universal Single-Copy Orthologs (BUSCO) (version 4.0.5) (11) with a stramenopile set of 100 single-copy orthologs (stramenopiles_odb10), and 83% of those orthologs were completely captured.

### Data availability.

The genome sequence of *T. laevis* has been deposited at the DDBJ under the accession numbers BLQM01000001 to BLQM01000902. Raw data are available in the DDBJ Read Archive with the accession numbers DRR213786 to DRR213791, DRR213793 to DRR213797, and DRR214684 to DRR214685.
